# Structural biology: A golden era

**DOI:** 10.1371/journal.pbio.3002187

**Published:** 2023-06-29

**Authors:** Oliviero Carugo, Kristina Djinović-Carugo

**Affiliations:** 1 Department of Chemistry, University of Pavia, Pavia, Italy; 2 Department of Structural and Computational Biology, Max Perutz Labs, University of Vienna, Vienna, Austria; 3 European Molecular Biology Laboratory (EMBL), Grenoble, France; 4 Faculty of Chemistry and Chemical Technology, Department of Biochemistry, University of Ljubljana, Ljubljana, Slovenia

## Abstract

The past two decades have seen a dramatic transformation take place in the field of structural biology. This Perspective looks back at the changes that have taken place and the promise that protein structure prediction algorithms hold for the future.

## Teaser

In the past 2 decades, structural biology has transformed from a single technique used on single proteins to a multimodal integrative approach. Recently, protein structure prediction algorithms have opened new avenues to address challenging biological questions.

This article is part of the *PLOS Biology* 20th Anniversary Collection.

Information on the 3D structure of biological macromolecules (proteins, nucleic acids, and complex macromolecular assemblies) is essential for the mechanistic understanding of their function, underlying cellular processes, and involvement in diseases, as well as for drug discovery. Structural biology employs a variety of techniques such as X-ray crystallography, nuclear magnetic resonance spectroscopy, cryo-electron microscopy (cryo-EM), small angle X-ray scattering and computer modelling to determine the 3D structure of biological macromolecules at the atomic level. The information generated from structural biology research has applications in many areas beyond structural biology itself, such as medicine, biotechnology, and agriculture.

The first protein structure (for myoglobin) was published in 1958 [1]. This endeavor took more than 20 years and required the development of a new method for protein crystallography, the isomorphous replacement method to solve the phase problem. Each step was manual, laborious, and time-consuming and involved the isolation of a protein of interest from native sources, its crystallization, collection of diffraction data on photographic films, and computational analysis on computers with a fraction of the processing power of a typical modern mobile phone. In 1971, the Protein Structure Database (PDB) was established as the central archive for experimentally determined macromolecular structure data. PDB pioneered data sharing practices at a time when this was still foreign to the vast majority of other scientific disciplines. In 1991, 20 years later, PDB included 684 entries, but since then, there has been an exponential increase in the number of structures deposited.

To date, more than 206,000 structures of biological macromolecules have been experimentally determined, with the rapid increase due to technological innovations in all major structural biology techniques, including improvements in instrumentation, analytical software, robot-assisted automation, and availability of high-brilliance synchrotron radiation and X-Ray Free-Electron Laser sources, enabling not only high-quality experiments on increasingly challenging systems but also time-resolved studies. New magnet technology has allowed access to magnetic fields above 1 GHz, and coupling of nuclear spin excitation with methodologies for increasing the transfer of polarization has boosted signal-to-noise by a few orders of magnitude, enabling studies of large systems as well as time-resolved structural studies. Furthermore, recombinant DNA technologies reduced the first bottleneck in the structure determination pipeline, which was the availability of pure, soluble, and homogenous protein in large quantities. Crystallization, the second bottleneck, has been miniaturized and automated.

Many technological advances have resulted from structural genomics consortia, which were formed at the turn of the millennium as a response to large-scale genome sequencing projects and aimed at determining the 3D structure of every protein encoded by a given genome. In the past decade, advances in instrumentation and software have sparked a “resolution revolution” in cryo-EM [2], making it possible not only to achieve resolutions comparable to X-ray crystallography but also to conduct structural studies in intact cells. Advances in experimental methodologies have transformed structural biology into a multiscale and multimodal approach, which covers a wide range of sizes, resolutions, and timescales, by combining complementary approaches in an integrative manner.

One of the most difficult problems in structural biology is protein structure prediction, also known as modelling. Computational approaches, such as homology modelling, fold recognition (threading), docking, molecular dynamics, and ab initio methods, have been developed in parallel with experimental structure determination techniques to predict the 3D structure of macromolecules, as well as their dynamics and interactions. There are two main approaches for modern protein modelling: template-based and neural network (deep learning)-based. Template-based modelling, including fold recognition and homology modelling, uses available resembling structures as templates to generate structure models and has been used for decades, as implemented in the software suites Modeller and Phyre2. Some deep learning methods have been used to aid template-based modelling to produce better results [3]. In 2021, AlphaFold2 and RoseTTAFold were the first to use deep learning approaches [4,5] for protein modelling. This brought about a sensational breakthrough in protein structure prediction and ignited a new revolution in structural biology.

The Protein Data Bank was the source of the training data for Alphafold2, which at the time consisted of more than 170,000 protein structures. DeepMind and the EMBL’s European Bioinformatics Institute partnered to create AlphaFold DB to make structure predictions freely available to the scientific community [6]. The most recent database release comprises approximately 200 million entries with extensive coverage of the UniProt database and has transformed the way experimental structural biology is performed and democratized access and use of information about protein 3D structure. Importantly, the reliability of models is estimated at the level of individual amino acids by the predicted local distance difference test. This enables the user to detect potentially uncertain structural regions, such as those caused by conformational disorder. Excellent follow-up advances in the prediction of protein–protein interactions and automatization of pipelines have been made with AlphaFold-Multimer [7] and AlphaPullDown [8], respectively, as well as on generation of models loaded with their ligands with AlphaFill [9], which “transplants” small molecules and ions from experimentally determined structures to predicted protein models.

Not surprisingly, these predictions have been challenged on numerous fronts. Some consider them suboptimal because they are based on statistics rather than first physicochemical principles, while others say that, at least for the time being, the accuracy of experimental structures cannot be reached. Careful comparison of AlphaFold2 models with a recently redetermined set of crystal structures revealed that the differences are greater than those between high-resolution structure pairs containing the same components but determined in different space groups [10]. Due to the nature of the prediction and the available training dataset, AlphaFold2 does not accurately model protein aggregation, macromolecular complexes (in particular protein–nucleic acids interactions, macromolecule–ligand interactions, and structural rearrangements triggered by, e.g., ligand binding), or external factors (e.g., pH, temperature, or conformationally dynamic systems), and further developments will be required (Box 1). Furthermore, although the effect of missense mutations on the thermodynamic stability of proteins can be predicted equally well by using experimental structures or AlphaFold2 models [11], AlphaFold2 currently does not appear to be suitable for predicting the structure of mutated proteins [12].

**Box 1. Avenues to further develop deep learning–based protein structure modelling**

Deep learning–based protein structure modelling is an extremely potent tool that has already had a major impact on structural biology. The following are potential future avenues for development of deep learning–based protein structure modelling tools such as Alphafold2, which should be guided by a better understanding of the first physiochemical principles that govern macromolecular folding and stability.

- Prediction of nucleic acids and other non-proteinaceous biological macromolecules.
- Prediction of macromolecular interactions: protein–protein, protein–nucleic acid, and protein–ligand.
- Prediction of structural ensembles of intrinsically disordered proteins and dynamic systems. While AlphaFold2 currently predicts static structures, proteins are dynamic and this is a feature intrinsically related to their function. Furthermore, intrinsically disordered proteins do not adopt a folded 3D structure and can be described as ensembles.
- Prediction of folding pathways and protein stability.
- Prediction of the impact of mutations on fold integrity, stability, and function.
- Prediction of posttranslational modifications, which can have a significant impact on protein structure and function.
- Applications in drug design, being part of drug design pipelines to identify new drug targets and design more effective medications.

Despite the aforementioned shortcomings, the availability of a realm of 3D models of proteins has sparked a revolution in the design and execution of experimental structural biology projects. In the absence of homologous experimental structures, AlphaFold2 models can be used to bootstrap structure determination using X-ray crystallography (as reliable molecular replacement search models), cryo-EM/ET (structural models fitting electron density maps and interpretation of low-resolution regions), or for modelling small-angle X-ray scattering-derived molecular envelopes. A fine example of the successful use of AlphaFold2 models is the integrative structural biology analysis of the nuclear pore complex [13]. Furthermore, AlphaFold-Multimer and AlphaPullDown are being used successfully for interaction discovery, which requires experimental validation as the subsequent step. AlphaFold2 predictions, therefore, provide a solid foundation for generating hypotheses about molecular mechanisms of action, enable the design of experiments with specific expected outcomes, and permit the investigation of complex systems that would be difficult or impossible to study through conventional methods.

Looking to the future, there is a growing recognition that mechanistic structural information on molecular machines is best generated in the context of intact cells in order to understand how they function in a complex native environment. This will require an integrative structural biology approach, which necessitates not only the improvement and expansion of structural training sets for superior modelling but also the integration of diverse data from complementary approaches such as proteomics and proximity-sensitive super-resolution imaging, with the ultimate goal of generating an integrated multiscale whole-cell model. Moreover, biological macromolecules function through conformational rearrangements, and a significant portion of the proteome, estimated to be over 30% in eukaryotes, is intrinsically disordered, lacking a fixed or ordered 3D structure, and represents the “dark matter” of biology that operates on the edge of chaos. A single structure is insufficient for comprehending the function of enzymes and molecular machines, nor does it describe spatially and temporally heterogeneous structural ensembles. The pursuit of 4D structures of complexes throughout a functional cycle, where the fourth dimension is a general reaction coordinate often interpreted as time and of the structure–function continuum underlying the intrinsic disorder, is a growing trend in structural biology.

Integrative structural biology has entered its golden age, capitalizing on a combination of multiscale methodologies combined with deep learning–based approaches such as AlphaFold2, which are enabling transformative science in structural biology and beyond, and will likely have a significant role in shaping the field’s future.
